# Evidence for Selection on Mitochondrial OXPHOS Genes in the Mediterranean Killifish *Aphanius fasciatus* Valenciennes, 1821

**DOI:** 10.3390/biology13040212

**Published:** 2024-03-25

**Authors:** Anna Maria Pappalardo, Giada Santa Calogero, Radek Šanda, Marta Giuga, Venera Ferrito

**Affiliations:** 1 Department of Biological, Geological and Environmental Sciences, Section of Animal Biology “M. La Greca”, University of Catania, Via Androne 81, 95124 Catania, Italy; giada.calogero@phd.unict.it (G.S.C.); marta.giuga@phd.unict.it (M.G.); 2 National Museum of the Czech Republic, Václavské Náměstí 68, 115 79 Prague, Czech Republic; radek.sanda@nm.cz; 3 Institute for the Study of Anthropic Impact and Sustainability in the Marine Environment (IAS-CNR), Via De Marini 6, 16149 Genova, Italy

**Keywords:** positive selection, OXPHOS genes, killifish

## Abstract

**Simple Summary:**

The old world killifish *Aphanius fasciatus* is a typical inhabitant of transition waters along the coasts of the central and eastern Mediterranean Sea, where it lives in environments (salt ponds, estuaries, and coastal lagoons) with large variations in salt, oxygen, and temperature. Therefore, *A. fasciatus* could be an interesting non-model species to be studied to evaluate the response of the mitochondrial oxidative phosphorylation (OXPHOS) genes to the variations and extremes of environmental factors. In this study, the sequences of three OXPHOS genes were analyzed in six populations of *A. fasciatus* to detect mutations and sites subject to selection. The results indicate that mutations were detected in two genes of the Greek population. Moreover, positively selected sites were also found. The information we obtained from the mitochondrial DNA sequences of *A. fasciatus* adds to the growing data on selective pressure acting on mitochondrial DNA. These results should be explored from the perspective of the local adaptation of a species highly tolerant to wide fluctuations of environmental parameters and should be supported using experimental evidence to better understand the interplay between historical climatic events and local adaptation and how each of them contributes to shaping the genetic structure of this species.

**Abstract:**

Mitochondrial oxidative phosphorylation (OXPHOS) genes are a system subject to selection under determined environmental constraints despite a neutral evolution model that has long been hypothesized for the mitochondrial genome. In this study, the sequences of *ND1*, *Cytb*, and *COI* OXPHOS genes were analyzed in six populations of the eurythermal and euryhaline killifish *A. fasciatus*, to detect non-synonymous mutations leading to amino acid changes and to check whether selection acted on them using tests of recombination and selection. The results indicate a high *COI* and *Cytb* gene diversity and a high percentage of private haplotypes in all populations. In the Greek population, non-synonymous nucleotide substitutions were observed in the N-terminal region of *COI* and *Cytb*. Positively selected sites were also found. The information we obtained from the mitochondrial DNA sequences of *A. fasciatus* adds to the growing data on selective pressure acting on mitochondrial DNA in non-model species. These results should be explored from the perspective of the local adaptation of eurythermal and euryhaline species and supported using experimental evidence to better understand the interplay between historical climatic events and local adaptation and how each of them contributes to shaping the genetic structure of this species.

## 1. Introduction

The mitochondrial oxidative phosphorylation (OXPHOS) genes have been studied in recent decades as a system that is subject to selection under certain environmental constraints [1,2,3] despite the fact that (i) a high level of preservation of its functions exists among eukaryotes and (ii) a neutral evolution model has long been hypothesized for the mitochondrial genome. This highly conserved system involved in the cellular respiration process for ATP production engages five main multimeric protein complexes (I–V) that together constitute the core of the OXPHOS system. The protein subunits that make up four (complexes I, III, IV, and V) of the five complexes of the electron transport chain are encoded by 13 mitochondrial genes together with several nuclear genes. Focusing on the function of these four complexes, the seven subunits of the NADH dehydrogenase complex I couple the oxidation of NADH to NAD+ with the translocation of 4 protons to the inner mitochondrial space. The cytochrome b (Cytb) subunit of the Cytochrome bc1 complex III is involved in the transfer of electrons from quinol to cytochrome c, which in turn transfers them to the inner mitochondrial membrane. The Cytochrome c Oxidase (COI) complex IV, including the COI, COII, and COIII subunits, generates water molecules from electrons carried by cytochrome c and molecular oxygen. Finally, the ATP synthase complex V, consisting of ATP6 and ATP8 subunits, synthesizes ATP via phosphorylation of ADP by exploiting the electrochemical energy generated using the proton gradient.

All these proteins are located in the inner mitochondrial membrane, and their function is highly conserved. However, it has been assessed that in order to meet the diverse metabolic needs of organisms via energy production, the OXPHOS genes are easily subjected to various selective pressures that promote adaptive changes. Indeed, the analysis of many of these genes has proven successful in identifying the traces of purifying (negative) and adaptive (positive) selection in various organisms associated with their adaptation to particular environmental factors. Evidence of positive selection on metabolism-related genes of mitochondrial DNA (mtDNA) has recently been detected for example in mammalian species of the order Cetartiodactyla [4], in species of the genus *Lepus* [5], in gentoo penguin *Pygoscelis papua* [3], in Polyplacophora Molluscs [6], in Orthoptera insects [7], in *Drosophila melanogaster* [1]. In all cited cases, environmental and/or climatic changes appear to drive the species adaptation.

With regard to fishes, according to [8], changes in mtDNA evolution are generally driven by selective pressures to increase aerobic capacity or to adapt to changes in environmental temperature. In this context, the positive selection in the *ND1*, *ND3*, and *ND4* genes detected in northern populations of *Salmo salar* has been hypothesized to be linked to the request for higher aerobic capacity at low temperatures [9]. On the other hand, it has been demonstrated that thermal environmental stress deeply affects cellular metabolism via the shift of enzyme functions, reduction in OXPHOS efficiency, increased inner membrane proton leakage, and decreased ATP production, all of which adversely affect cellular and lastly, organismal survival [10]. In addition, mitochondrial respiration is particularly dependent on habitat temperature in ectotherms, as they depend largely upon environmental radiation to thermoregulate themselves, and it has been demonstrated that environmental temperatures above the optimal temperature for each individual species could impair mitochondrial function [11]. However, it is remarkable that fish and mollusk species living in coastal marine environments, which are naturally subject to rapid changes in environmental variables, are able to cope with a wide spectrum of temperature, oxygen, and salinity levels via a rapid modulation of the mitochondrial electron transport system [12,13].

The old world killifish *Aphanius fasciatus* is a typical inhabitant of transition waters along the coasts of the central and eastern Mediterranean Sea and shows peculiar biological traits. During the breeding season, the species lays large demersal eggs that remain attached to the vegetation; it lacks a larval stage and shows a rapid turnover due to the short generation time and the high reproductive rate. Adults are highly mobile but also show high site fidelity because they only move from one site to another adjacent site during heavy rain events that cause the coastal lake to overflow. Therefore, the gene flow between populations is very limited. Due to its strong euryhalinity and eurythermy, it is adapted to live in environments (salt ponds, estuaries, and coastal lagoons) with large salt, oxygen, and temperature variations where it goes through its entire life cycle. In particular, this species occupies shallow aquatic environments that can be easily heated locally by solar insolation, where it experiences wide daily and seasonal fluctuations in water temperature along the Mediterranean coast. Therefore, *A. fasciatus* could be an interesting non-model species to be studied to evaluate the response of the OXPHOS genes to the variations, even extreme ones, in environmental factors.

Based on the above considerations, in this study, we analyze the sequences of *ND1*, *Cytb,* and *COI* genes in six populations of the killifish *A. fasciatus* sampled along the Italian, Albanian, and Greek coasts. The aims are: (i) to explore the nucleotide sequences of the three OXPHOS mitochondrial genes to detect the presence of mutations leading to non-synonymous amino acid changes and (ii) to check whether selection acted on them using tests of recombination and selection based on different models of evolution.

## 2. Materials and Methods

### 2.1. Sampling Sites

**Figure 1:**
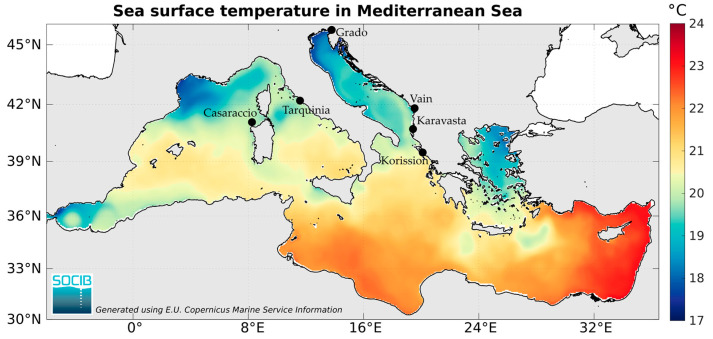
Heatmap of annual mean sea surface temperature and sampling locations (black dots) of the killifish *Aphanius fasciatus*.

Six populations of the killifish *A. fasciatus* were sampled along the coast of Italy (Grado, Tarquinia, and Casaraccio lagoons), Albania (Vain and Karavasta lagoons), and Greece (Korission lake) coasts (Figure 1, Table 1). Most of these coastal lagoons are not very deep, with a complex hydrographical network including tidal flats, tidal channels, and subtidal zones; the Korission lagoon is the deepest one, with a maximum depth of 2 m.

Being shallow water bodies, their temperatures can be heavily influenced by air temperature: in any case, there is a significant temperature range between summer and winter. For example, in Grado, Karavasta, and Korission, temperature values can range from 6–7 °C to 26–30 °C [14,15,16], while in other cases, temperatures can be even higher up until 35–36 °C for Tarquinia [17].

Another important parameter concerns the salinity of the water, as it depends on the degree of freshwater inputs, which are mostly related to the rainfall amount or drainage channels. In some cases, there is a gradient existing within the water mass itself, for example, in Grado and Tarquinia [17,18], and this will regulate the distribution of fish fauna and macrozoobenthic species; while in other cases the salinity changes between seasons, being very much higher in summer, for example in Karavasta [19], Casaraccio [20] and Korission [16] so that they can be characterized by hypersaline water.

As for the trophic state, among the ones taken into consideration, the Casaraccio lagoon is the one with lower organic and mud contents because it has no significant anthropogenic impacts and it has been able to preserve the highest degree of naturalness [20] and also the Grado lagoon does not exhibit significant effects of eutrophication [14]. Between the sites taken into account, the Vain lagoon is classified as eutrophic, because it presents green and murky water, with higher amounts of nutrients and algae; this was further demonstrated using the analysis of chlorophylls content which exhibited high values, showing that the high content of Cyanophyceae is linked to a higher eutrophication level [21].

Six populations of the killifish *A. fasciatus* were sampled along the Italian, Albanian, and Greek coasts for a total of 116 individuals (Figure 1).

### 2.2. DNA Extraction

Genomic DNA was extracted using the DNeasy Blood and Tissue Kit (QIAGEN, Hilden, Germany) following [22] (Table 1).

### 2.3. Amplification and Sequencing of the OXPHOS Target Genes

Three mitochondrial genes, each encoding different subunits of the OXPHOS complex I (*ND1* gene), complex III (*Cytb* gene), and complex IV (*COI* gene), were amplified using specific primer pairs reported in Table 2. PCR amplification mixes for each target gene were prepared following [23] and using different thermal profiles (Supplementary Material Figure S1). All obtained amplicons were sequenced at Eurofins Genomics (https://eurofinsgenomics.eu, accessed on 3 September 2023), and sequences were submitted to the GenBank database (Supplementary Material Tables S1–S3).

### 2.4. Data Analysis

Multiple sequence alignments and editing were performed in MAFFT [27] and BioEdit (http://www.mbio.ncsu.edu/bioedit/bioedit.html, accessed on 15 November 2023), respectively, and mitochondrial haplotypes were identified for each gene. Descriptive genetic diversity indices such as nucleotide (π), haplotype diversity (*h*), and number of haplotypes (H) were estimated as implemented in DNAsp 6 [28] for each gene and population. Elaboration of the diversity indexes of the OXPHOS target genes was performed in GraphPad Prism 8.3.0 software (https://www.graphpad.com, accessed on 17 November 2023). Relationships among the *COI*, *Cytb*, and *ND1* haplotypes identified in each population were depicted with the median-joining (MJ) method in Network 10.2 (Fluxus-engineering.com, accessed on 15 March 2024). A Maximum Likelihood (ML) tree was generated in MEGA X software [29] for each examined gene using the species *Apricaphanius iberus* as an outgroup [Genbank Acc. N.: KJ552729 (*COI*), KU174389 (*Cytb*), EF640857 (*ND1*)].

### 2.5. Recombination and Selection Tests

The Genetic Algorithms for Recombination Detection (GARD) (HyPhy package, accessed at www.datamonkey.org, accessed on 20 November 2023) was used to check for the presence of mitochondrial *COI*, *Cytb*, and *ND1* recombinants. A preliminary one-tailed Z-test in MEGA X [29] was performed to test the presence of selection for each gene dataset. Furthermore, three codon models were used to estimate codons under positive or purifying selection: FEL (Fixed Effects Likelihood) [30], FUBAR (Fast, Unconstrained Bayesian Approximation for Inferring Selection) [31], and MEME (Mixed effects model of evolution) [32]. Sites with *p*-values below 0.05 for FEL and MEME, as well as sites with posterior probability higher than 0.9 for FUBAR, were all considered as being under selection [33].

### 2.6. Protein Structure Analysis

To show the spatial position of sites under positive selection in a 3-dimensional space, we used the SWISS-MODEL server (https://swissmodel.expasy.org/, accessed on 10 December 2023) with default parameters [34]. In order to visualize the structural position of positively selected sites, the secondary structures of the three protein models were predicted using the PSIPRED server (http://bioinf.cs.uci.ac.uk/psipred/, accessed on 13 December 2023) [35], and the positions of transmembrane helices were predicted using the MEMSAT-SVM server (http://bioinf.cs.uci.ac.uk/psipred, accessed on 13 December 2023).

## 3. Results

### 3.1. Gene Sequences Analysis and Distribution of Genetic Variation

**Figure 2:**
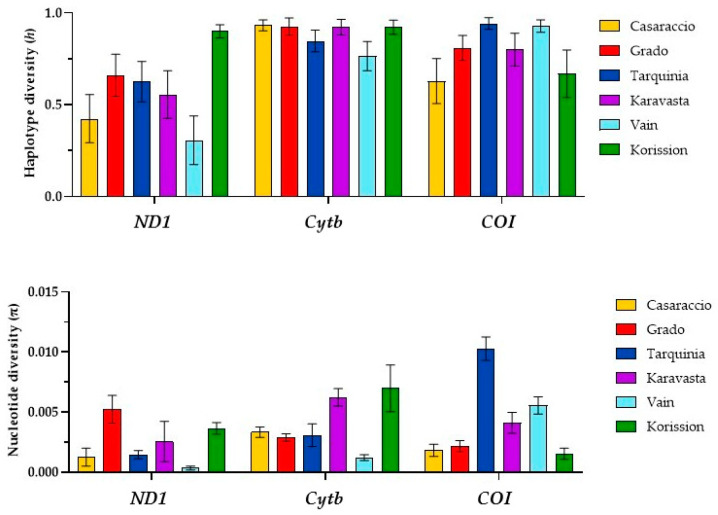
Plots of haplotype (*h*) and nucleotide (*π*) diversity indexes of the killifish *Aphanius fasciatus* OXPHOS target genes. Error bar indicates standard deviation.

The 116 individuals of killifish *A. fasciatus* were processed to amplify and sequence the three targeted genes. Sizes of the fragments utilized varied across genes, specifically *COI* (669 bp), *Cytb* (1125 bp), and *ND1* (909 bp). All sequences produced 55 haplotypes for the *COI* gene (GenBank Acc. N. OR064287-OR064341), 56 for *Cytb* (GenBank Acc. N. OR134774-OR134829), and 30 for *ND1* (GenBank Acc. N. OR097693-OR097722) (Supplementary Material Tables S1–S3). As expected, genetic diversity indexes varied among analyzed genes (Figure 2).

Low values of haplotype diversity were found in the *ND1* gene for all populations with the exception of Korission, where *h* was 0.900 (Figure 2); *h* values ranging from 0.629 to 0.932 were found in *COI* e *Cytb* genes for all populations. In addition, low values of nucleotide diversity were observed for the *ND1* gene with the exception of the Grado population, while the highest *π* value was found in the *COI* gene of the Tarquinia population.

**Figure 3:**
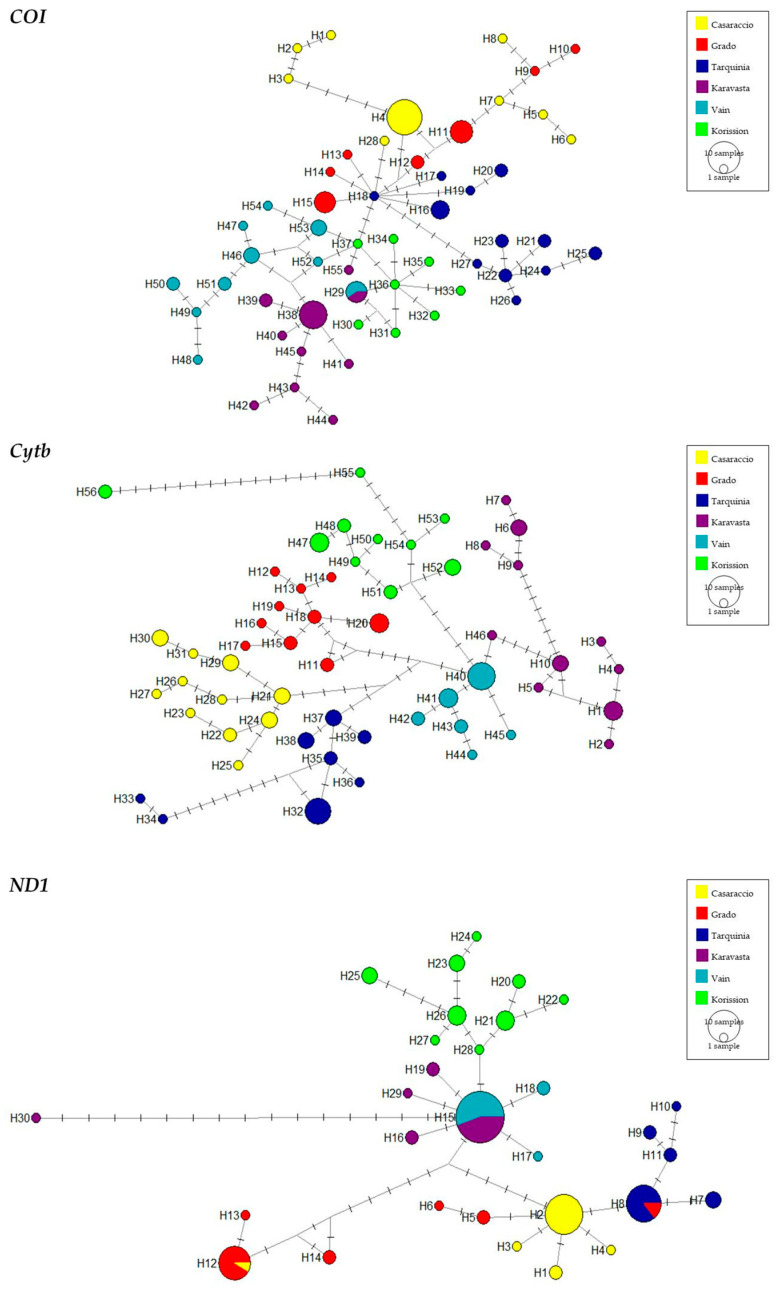
Median-joining network for *COI, Cytb*, and *ND1* sequences of the killifish *Aphanius fasciatus*. The networks mostly illustrate haplotype diversity and relationships since circle sizes are representative of sample size. Crossbars indicate single substitutions.

The haplotype networks (Figure 3) show only a few shared haplotypes for the *COI* and *ND1* genes (1 shared haplotype between Karavasta and Vain and three shared haplotypes) and none for the *Cytb* gene. Accordingly to the *COI* and *Cytb* haplotype networks, there are many private haplotypes for each population represented by a few individuals, whereas, for the *ND1* gene, the number of identified haplotypes is lower with the presence of more shared haplotypes, namely H8, H12, and H15.

**Figure 4:**
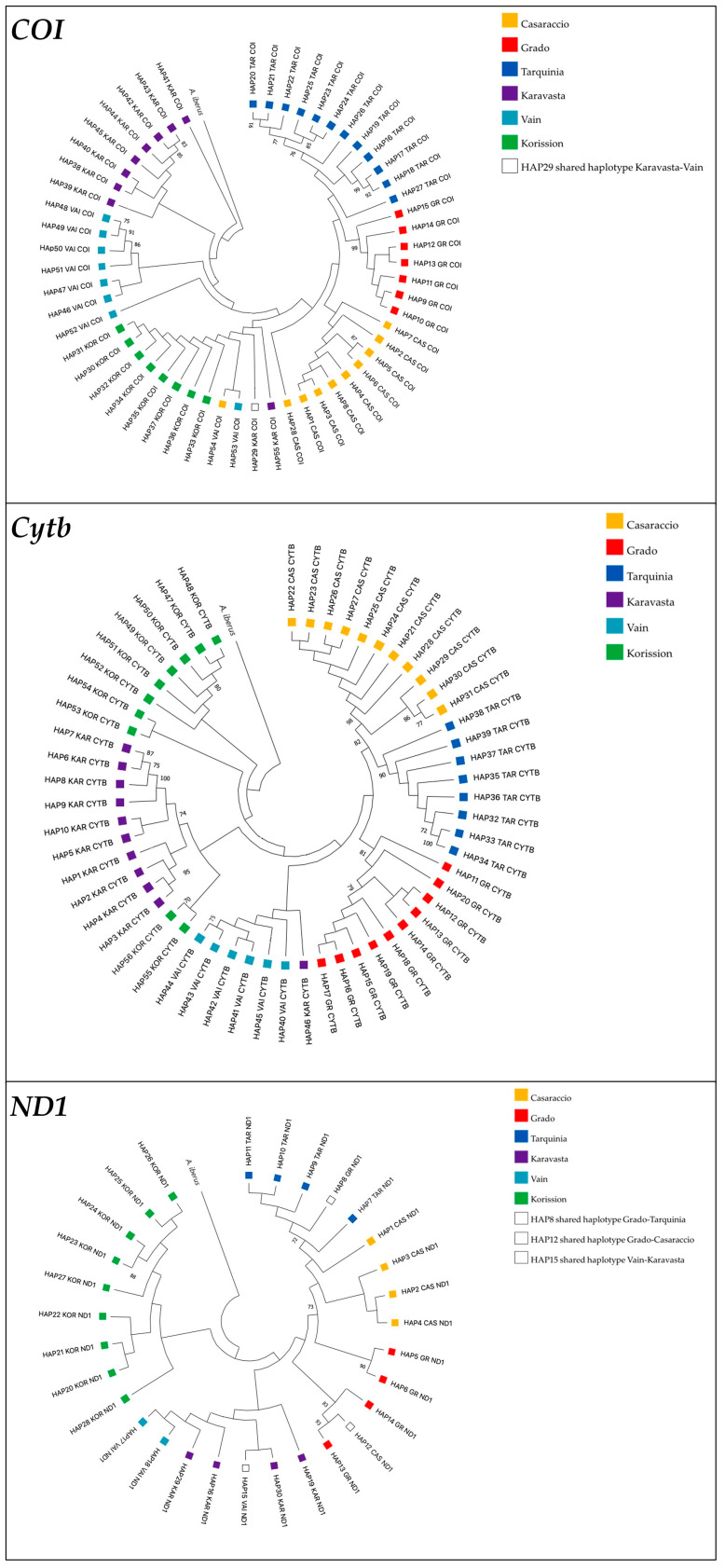
Maximum likelihood trees showing phylogenetic relationships among killifish *Aphanius fasciatus* sequences based on *COI*, *Cytb*, and *ND1* genes. Values at nodes indicate bootstrap support. Only values higher than 70% are shown. The topology is rooted in the Spanish toothcarp *Apricaphanius iberus*.

The ML trees constructed using *COI*, *Cytb*, and *ND1* sequences were reported in Figure 4.

In line with the network, the *COI* ML tree shows that all populations have private haplotypes with the exception of Karavasta and Vain populations (Albania), which shared the HAP29, while no shared haplotypes were found in the *Cytb* ML tree. The *ND1* ML tree shows that there are three shared haplotypes: HAP8, present in Italian populations of Grado and Tarquinia; HAP12, present in the Italian populations of Grado and Casaraccio; and HAP15, found in the Albanian populations of Vain and Karavasta (Figure 4).

### 3.2. Evidence of Selection Signals in Mitochondrial Genes

No evidence for recombination was found in *COI* and *ND1* datasets with GARD. For the *Cytb* dataset, GARD showed a recombination break, so the two partitions were analyzed separately. The null hypothesis of strict neutrality was rejected by the Z test in favor of the alternative hypothesis of positive selection in the three analyzed genes.

For the *COI* gene, eight codons were under purifying selection, and two codons were under positive selection (codons 9 and 111). In particular, the MEME method detected episodic positive selection for codon 9, while FUBAR revealed pervasive positive selection for codon 111. For the *Cytb* gene, five codons were under purifying selection, and four codons were under positive selection. Codons 318 and 375 were under episodic positive selection with the MEME method, while FUBAR detected pervasive positive selection for codons 9 and 320. For the *ND1* gene, eight codons were under purifying selection, and only codon 300 resulted in positive selection with the FUBAR method (Table 3).

### 3.3. Non-Synonymous Changes Analysis

Nucleotide substitutions leading to non-synonymous aminoacidic changes were observed in the Korission population in the *COI* (223 aa sequence long) and *Cytb* (375 aa sequence long) genes. For the *COI* gene, a nucleotide substitution in position 64 from G to A led to the non-synonymous aminoacidic change from alanine (A) to threonine (T) in codon 22.

For *Cytb* gene: (i) a nucleotide substitution in position 8 from A to C resulted in a non-synonymous aminoacidic change from asparagine (N) to threonine (T) in codon 3; (ii) nucleotide substitution in position 11 from C to A led a non-synonymous aminoacidic change from proline (P) to histidine (H) in codon 4; (iii) a nucleotide substitution in position 17 from C to T leading a non-synonymous aminoacidic change from proline (P) to leucine (L) in codon 6 and (iv) a nucleotide substitution in position 21 from T to A resulting in an aminoacidic change from phenilalanine (F) to Leucine (L) in codon 7. For the *ND1* gene (303 aa sequence long), only synonymous changes were observed (Table 4).

**Figure 5:**
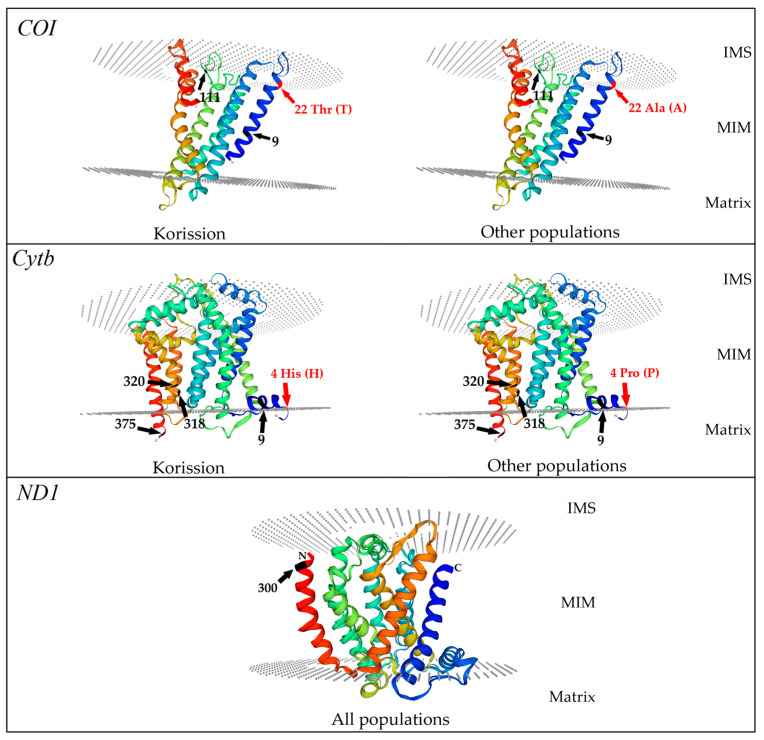
Mapping of the positively selected sites (black arrow) and the non-synonymous aminoacidic changes (red arrow) in the 3-dimensional obtained models for killifish *Aphanius fasciatus* COI, Cytb, and ND1 proteins. The mitochondrial inner membrane (MIM) is delineated with the two dashed disks. IMS: intermembrane space; Thr (T), threonine; Ala (A) alanine; His (H), histidine; Pro (P), proline. N, amino-terminal tail; C, carboxy-terminal tail. Numbers and arrows indicate codon positions.

Mapping of the non-synonymous aminoacidic changes on the 3-dimensional structure of the obtained models for the examined mitochondrial proteins is shown in Figure 5.

### 3.4. 3D Models of COI, Cytb, and ND1 Fragment Proteins

In order to understand the position of the sites under positive selection, three protein models for each mitochondrial subunit were generated (Figure 5).

In the COI barcode region, the non-synonymous mutation Ala22Thr is located in the N terminal tail, where we also found codon 9 under episodic positive selection, while codon 111 under pervasive positive selection is placed in the coil of the protein facing towards the intermembrane space (IMS) (Figure 5).

In the Cytb segment, four non-synonymous aminoacidic mutations (codons: 3, 4, 6, 7) are sited in the N-terminal tail, while the codon change in 111 positions is located in the third transmembrane α-*h*elix. We found one positively selected codon (9) in the N-terminal tail, while codons 318 and 320 under positive selection are in the seventh α-*h*elix and codon 375 in the C-terminal tail (Figure 5).

In the ND1 region, the only codon (300) found under positive selection is located in the C-terminal tail (Figure 5).

## 4. Discussion

The sequence analysis of three OXPHOS genes, *ND1*, *Cytb*, and *COI*, was carried out on six populations of the Mediterranean killifish *A. fasciatus*, sampled in coastal ponds along a North–South thermal gradient: the Italian population of Grado was the northernmost, and the Greek one of Korission was the southernmost. The results indicate that a high haplotype and nucleotide diversity characterizes the *COI* and *Cytb* genes and that all three genes show a high percentage of private haplotypes. Very peculiar in this regard seems to be the Greek population of Korission, which in the ML trees does not share any haplotype with the other populations for each gene examined. Overall, these results confirm those of previous studies on the genetic structure of *A. fasciatus* using various mitochondrial molecular markers [35,36,37,38,39,40,41,42]. They can be explained by considering the peculiar biological traits of this species that trigger the divergence among populations. In particular, the absence of larval stages and the low dispersal potential of adults promote high fidelity to the site. In addition, the adaptation to naturally fragmented environments with large variations in temperature (from 4 to 40 °C) and salinity (from freshwater to 40‰ hypersaline water bodies) promotes the selection of population-specific gene pools. For these reasons, *A. fasciatus* is an ideal species to study microevolutionary processes driven by adaptation to the environment. In this context, although the assumption of neutrality of mitochondrial DNA has long been advocated, it is now demonstrated that mitochondrial genes coding for OXPHOS enzymes are easily subjected to selective pressure in variable environmental contexts since they are responsible for the metabolic performance of organisms. Therefore, several investigations have explored the nucleotide sequences of the OXPHOS mitochondrial genes to detect the presence of non-synonymous mutations leading to amino acid changes, eventually affecting the function of the encoded proteins under environmental constraints [5]. In our case, non-synonymous nucleotide substitutions leading to amino acid changes were observed in the Korission population of *A. fasciatus* in codon 22 of the *COI* gene and in codons 3,4,6 and 7 of the *Cytb* gene. All these mutations are located in the N-terminal tail of both proteins, and while the main part of them entailed the substitution of amino acids with the same polarity, only the amino acid substitution at codon 22 of COI protein and that at codon 4 of the Cytb protein resulted in a change of the physiochemical properties of the amino acids. In particular, the non-polar alanine (A) was substituted by the polar uncharged threonine (T) in codon 22 of the COI protein. Alanine and threonine are both amino acids small in size, but while alanine has a side chain non-reactive, threonine has a fairly reactive hydroxyl group. Accordingly, while A is rarely involved in protein function, T is common in protein functional centers [43]. Looking at the nucleotide sequence of the *COI* barcode region examined by us, it is known that its discriminative power for species identification is based on the high variability of the third codon position of amino acids, but taking into account the amino acids deriving from changes in this position, it should be noted that about 72% of them are synonymous and do not lead to amino acid changes, while only 5% of changes in the first position are synonymous, proving that the amino acid sequence of COI is basically conserved as well as its function [44]. In our case, the nucleotide triplets encoding alanine (four codons) and threonine (four codons) amino acids differ only in the first position, which is G in alanine and A in threonine. Furthermore, our *COI* sequences perfectly overlap with those of 323 Actinopterygii analyzed by [44], and based on the degree of conservation of each amino acid position, alanine 22 should be highly conserved contrary to our observations.

Looking at Cytb protein, the non-polar proline (P) was substituted by the polar basic histidine (H) in codon 4. The proline side chain is very non-reactive, and it is rarely involved in protein-active or binding sites, while histidine is the most common amino acid in protein-active or binding sites [43]. It is noteworthy that the N-terminal tail of the Cytb protein belongs to the matrix domain, which is involved in a few functional activities. However, it has been highlighted that a few amino acid residues close to the N-terminal tail are implicated in the creation of the proton gradient [45,46]. The functional importance of amino-acid substitution and its effects on the physicochemical properties of the N-terminal region of the Cytb protein was also suggested by the results of the investigation by [47], which demonstrated that the substitution that introduces a threonine (polar) instead of isoleucine (non-polar) in the position 7 of the human Cytb, led the region surrounding site 7 to become more hydrophilic and more open and free to interact. Accordingly, we hypothesize that the substitution of amino acids we detected could have important functional implications, such as making the N-terminal region of the Cytb protein more hydrophilic.

The second aim of our investigation was to check whether selection acted on the three OXPHOS genes under consideration using recombination and selection tests based on different models of evolution. Notably, the main part of codons was found under purifying or negative selection, as expected for genes encoding proteins involved in important mitochondrial functions as they are OXPHOS enzymes. However, the search for sites that evolve under positive selective pressure yielded interesting results, especially for COI and Cytb proteins. In particular, codon 9 of the COI, again located in the N-terminal tail facing the mitochondrial inner membrane (MIM), was found to be under episodic positive selection detected using MEME, and codon 111, located in the coil of the protein facing towards the intermembrane space, under pervasive positive selection detected using FUBAR. For Cytb, codons 9 and 320 were found under pervasive positive selection, and codons 318 and 375 were under episodic positive selection. Apart from codon 375, located at the C-terminal tail within the matrix, all the remaining codons are located in the inner mitochondrial membrane. Finally, for *ND1*, codon 300, once again located in the N-terminal region, was found under pervasive positive selection. However, none of the *COI* and *Cytb* codons under positive selection were affected by non-synonymous substitutions.

Signatures of positive selection on OXPHOS genes have been found in several teleost species in response to the need to increase aerobic capacity or adapt to changes in environmental temperature. Positively selected sites occurring in the *COI* gene of Glyptosternoid fishes from the southeastern Tibetan Plateau and other OXPHOS genes were found to drive adaptation to the high-elevation environment. It has also been hypothesized that the interaction between positively selected sites of COX subunits could affect enzyme regulation and OXPHOS efficiency in Istiophoridae, a family of highly aerobic fish [48]. A signature of positive or directional selection was found in three sites of *Cytb* in *Sardina pilchardus* populations, and a correlation between the most frequent *Cytb* haplotype and the minimum sea surface temperature was detected [49]. Interestingly, the mitochondrial performance of the heart and brain, following thermal acclimation to extreme temperatures, in locally adapted subspecies of the Atlantic killifish *Fundulus heteroclitus*, highlighted a complex mitochondrial regulation in this deeply eurythermal species, with OXPHOS efficiency and reactive oxygen dynamics probably playing a pivotal role in thermal acclimation and local adaptation [50]. In addition, the electron transport system and OHPHOS enzymes have been indicated as promising targets to study the high capacity of mitochondrial regulation in intertidal organisms (mollusks, crustaceans, and fish) facing rapid changes in environmental parameters [13]. In our case, it is evident that the N-terminal region of COI and Cytb, which host both non-synonymous mutations leading to amino acid changes and codons under positive selection, could play an important functional role within the OXPHOS system. However, we did not explore the relationship between the sequence mutation and/or the positively selected sites detected and environmental parameters. In this respect, it should be taken into account that each *A. fasciatus* population may experience the same extreme environmental parameters in the transitional habitats in which they live, characterized by wide daily and seasonal fluctuations in temperature, dissolved oxygen, salinity, and other physicochemical parameters. Furthermore, we cannot overlook the peculiar biological characteristics of this Mediterranean killifish, which, on the one hand, favor the genetic isolation of populations and, on the other, make it highly resistant to the stressful conditions typical of the environments in which it lives.

It is noteworthy, however, that non-synonymous mutations are exclusively found in the Greek population of Korission, which is the southernmost of those we tested. In contrast, the sites subjected to selection remain as such even when the Greek population is removed from the analysis. Consequently, in order to understand which factors may individually or synergistically bring about functional changes in the proteins of the OXPHOS genes, experimental evidence must be sought to test the response of these mitochondrial genes to the variation of environmental parameters.

## 5. Conclusions

The information we obtained from the mitochondrial DNA sequences of *A. fasciatus* adds to the growing data on selective pressure acting on mitochondrial DNA in non-model species. Our results confirmed both the presence of non-synonymous amino acid changes, the functional implications of which remain to be explored, and of positively selected sites in *COI* and *Cytb* proteins. However, these results (i) should be explored from the perspective of the local adaptation of eurythermal and euryhaline species and (ii) should be supported via experimental evidence to better understand the interplay between historical climate events and local adaptation and how each of them contributes in shaping the genetic structure of this species.

## Figures and Tables

**Table 1:** Sampling sites of the killifish *A. fasciatus* populations. Numbers of specimens examined, N and number of private haplotypes (h) detected for each gene.

Country	Locality	Coordinates	*COI*	*Cytb*	*ND1*
Italy	Casaraccio Lagoon	40°55′01″ N 8°13′04″ E	21 (9)	20 (11)	21 (4)
	Grado Lagoon	45°40′44″ N 13°23′45″ E	17 (7)	10 (10)	19 (6)
	Tarquinia Salt works	42°14′47″ N 11°44′50″ E	20 (12)	20 (8)	20 (4)
Albania	Karavasta Lagoon	40°56′02″ N 19°29′08″ E	20 (10)	18 (11)	18 (4)
	Vain Lagoon	41°45′46″ N 19°34′54″ E	18 (9)	18 (6)	18 (3)
Greece	Korission Lagoon	39°26′45″ N 19°54′14″ E	18 (8)	18 (10)	20 (9)
	Total		114 (55 + 1) *	110 (56)	116 (27 + 3) **

* 55 private haplotypes and 1 shared, ** 27 private haplotypes and 3 shared.

**Table 2:** List of primers used in this study.

Gene	ID Primer	Sequence	Reference
*COI*	VF2_t1	5′-TGTAAAACGACGGCCAGTCAACCAACCACAAAGACATTGGCAC-3′	[24]
	FishR2_t1	5′-CAGGAAACAGCTATGACACTTCAGGGTGACCGAAGAATCAGAA-3′	
	M13_FW	5′-TGTAAAACGACGGCCAGT-3′	[25]
	M13_REV	5′-CAGGAAACAGCTATGAC-3′	
*Cytb*	L14724	5′-GTGACTTGAAAAACCACCGTTG-3′	[26]
	H15915	5′-CAACGATCTCCGGTTTACAAGAC-3′	
*ND1*	ND1_Aph_FW	5′-TGATTTTAGTACTTTATGCAATTATTA-3′	This study
	ND1_Aph_REV	5′-GTGGGGGGGCAAGCCAGA-3′	

**Table 3:** Positively and negatively selected sites in killifish *Aphanius fasciatus* mitochondrial genes estimated by FUBAR (# *bpp* ≥ 0.9; ## *bpp* ≥ 0.95; ### *bpp* ≥ 0.99) SLAC and MEME models (* *p* < 0.05; ** *p* < 0.01; *** *p* < 0.001).

Codon		6	7	8	9	20	34	72	111	152	222
Selection type		Purifying	Purifying	Purifying	Positive	Purifying	Purifying	Purifying	Positive	Purifying	Purifying
***COI***	FEL	*	*	*		*	*	*		*	
	FUBAR	#	#					#	#		##
	MEME				***						
Codon		9	31	144	159	243	311	318	320	375	
Selection type		Positive	Purifying	Purifying	Purifying	Purifying	Purifying	Positive	Positive	Positive	
***Cytb***	FEL		*	**	*	*					
	FUBAR	#	#	#	#	#	#		#		
	MEME							*		**	
Codon		5	70	78	183	223	225	244	300	302	
Selection type		Purifying	Purifying	Purifying	Purifying	Purifying	Purifying	Purifying	Positive	Purifying	
***ND1***	FEL	*		*			*			*	
	FUBAR	##	##	#	#	##	#	#	#	#	
	MEME										

**Table 4:** Non-synonymous codon change in the killifish *Aphanius fasciatus COI* and *Cytb* mitochondrial genes. In red, chemical feature changes.

**Gene**	**Population**	**Codon 22**				
***COI***	Grado	A (N-polar)				
	Tarquinia	A (N-polar)				
	Casaraccio	A (N-polar)				
	Vain	A (N-polar)				
	Karavasta	A (N-polar)				
	Korission	T (polar)				
**Gene**	**Population**	**Codon 3**	**Codon 4**	**Codon 6**	**Codon 7**	**Codon 111**
***Cytb***	Grado	N (polar)	P (N-polar)	P (N-polar)	L (N-polar)	V (N-polar)
	Tarquinia	N (polar)	P (N-polar)	P (N-polar)	F (N-polar)	V (N-polar)
	Casaraccio	N (polar)	P (N-polar)	P (N-polar)	F (N-polar)	I (N-polar)
	Vain	N (polar)	P (N-polar)	P (N-polar)	F (N-polar)	V (N-polar)
	Karavasta	N (polar)	P (N-polar)	P (N-polar)	L (N-polar)	V (N-polar)
	Korission	T (polar)	H (polar, basic)	L (N-polar)	L (N-polar)	V (N-polar)

A = Alanine, T = Threonine, N = Asparagine, P = Proline, H = Histidine, L = Leucine, F = Phenylalanine, V = Valine, I = Isoleucine.

## Data Availability

All sequences were deposited in GenBank, and Supplementary Material is available online.

## Supplementary Materials

The following supporting information can be downloaded at: https://www.mdpi.com/article/10.3390/biology13040212/s1, Figure S1: Thermal profiles for PCR amplification of *COI*, *Cytb*, and *ND1* genes; Table S1: *A. fasciatus COI* haplotypes detected in this study; Table S2: *A. fasciatus Cytb* haplotypes detected in this study; Table S3: *A. fasciatus ND1* haplotypes detected in this study.

## References

[1] Lajbner Z., Pnini R., Camus M.F., Miller J., Dowling D.K. (2018). Experimental Evidence That Thermal Selection Shapes Mitochondrial Genome Evolution. Sci. Rep..

[2] Lu Z., Liu T., Liu Y., Wang Y., Liu J., Liu B., Gong L., Liu L. (2023). Climate Adaptation and Drift Shape the Genomes of Two Eel-Goby Sister Species Endemic to Contrasting Latitude. Animals.

[3] Noll D., Leon F., Brandt D., Pistorius P., Le Bohec C., Bonadonna F., Trathan P.N., Barbosa A., Rey A.R., Dantas G.P.M. (2022). Positive Selection over the Mitochondrial Genome and Its Role in the Diversification of Gentoo Penguins in Response to Adaptation in Isolation. Sci. Rep..

[4] Mori S., Matsunami M. (2018). Signature of Positive Selection in Mitochondrial DNA in Cetartiodactyla. Genes Genet. Syst..

[5] Awadi A., Ben Slimen H., Schaschl H., Knauer F., Suchentrunk F. (2021). Positive Selection on Two Mitochondrial Coding Genes and Adaptation Signals in Hares (Genus *Lepus*) from China. BMC Ecol. Evol..

[6] Dhar D., Dey D., Basu S., Fortunato H. (2021). Insight into the adaptive evolution of mitochondrial genomes in intertidal chitons. J. Molluscan Stud..

[7] Li X.D., Jiang G.F., Yan L.Y., Li R., Mu Y., Deng W.A. (2018). Positive Selection Drove the Adaptation of Mitochondrial Genes to the Demands of Flight and High-Altitude Environments in Grasshoppers. Front. Genet..

[8] Dalziel A.C., Martin N., Laporte M., Guderley H., Bernatchez L. (2015). Adaptation and acclimation of aerobic exercise physiology in Lake Withefish ecotypes (*Coregonus clupeaformis*). Evolution.

[9] Consuegra S., John E., Verspoor E., De Leaniz C.G. (2015). Patterns of Natural Selection Acting on the Mitochondrial Genome of a Locally Adapted Fish Species. Genet. Sel. Evol..

[10] Iftikar F.I., MacDonald J.R., Baker D.W., Renshaw G.M.C., Hickey A.J.R. (2014). Could Thermal Sensitivity of Mitochondria Determine Species Distribution in a Changing Climate?. J. Exp. Biol..

[11] Sokolova I.M. (2023). Ectotherm Mitochondrial Economy and Responses to Global Warming. Acta Physiol..

[12] Richards J.G. (2011). Physiological, Behavioral and Biochemical Adaptations of Intertidal Fishes to Hypoxia. J. Exp. Biol..

[13] Sokolova I. (2018). Mitochondrial Adaptations to Variable Environments and Their Role in Animals’ Stress Tolerance. Integr. Comp. Biol..

[14] Acquavita A., Aleffi I.F., Benci C., Bettoso N., Crevatin E., Milani L., Tamberlich F., Toniatti L., Barbieri P., Licen S. (2015). Annual characterization of the nutrients and trophic state in a Mediterranean coastal lagoon: The Marano and Grado Lagoon (northern Adriatic Sea). Reg. Stud. Mar. Sci..

[15] Çomo E., Hasimi A., Murtaj B., Hoxhaj J., Lushaj B. (2018). Evaluation of Physic-Chemical Features of the Main Coastal Lagoons of Narta and Karavasta, in Albania. Online Int. Interdiscip. Res. J..

[16] Roselli L., Stanca E., Ludovisi A., Durante G., Souza J.S.D., Dural M., Alp T., Bulent S., Gjoni V., Ghinis S. (2013). Multi-scale biodiverity patterns in phytoplankton from coastal lagoons: The Eastern Mediterranean. Transitional Waters Bull..

[17] Bonifazi A., Galli S., Gravina M.F., Ventura D. (2023). Macrozoobenthos Structure and Dynamics in a Mediterranean Hypersaline Ecosystem with Implications for Wetland Conservation. Water.

[18] Ferrarin C., Umgiesser G., Bajo M., Bellafiore D., De Pascalis F., Ghezzo M., Mattassi G., Scroccaro I. (2010). Hydraulic zonation of the lagoons of Marano and Grado, Italy. A modeling approach. Estuar. Coast. Shelf Sci..

[19] Koto R., Bani A., Skuka N. (2014). Physico-Chemical Characteristics and Heavy Metal Contents of Water from Karavasta lagoon, Albania. Albanian J. Agric. Sci..

[20] Gravina M.F., Cabiddu S., Como S., Floris A., Padedda B.M., Pusceddu A., Magni P. (2020). Disentangling heterogeneity and commonalities in nanotidal Mediterranean lagoons through environmental features and macrozoobenthic assemblages. Estuar. Coast. Shelf Sci..

[21] Bacu A., Babani F., Malollari I. (2011). A comparative study on the efficiency of use of different physical and biological parameters for the evaluation of the level of trophy in the lagoon system of Kune-Vain, Albania. J. Environ. Prot. Ecol..

[22] Pappalardo A.M., Gonzalez E.G., Tigano C., Doadrio I., Ferrito V. (2015). Comparative Pattern of Genetic Structure in Two Mediterranean Killifishes *Aphanius fasciatus* and *Aphanius iberus* Inferred from Both Mitochondrial and Nuclear Data. J. Fish. Biol..

[23] Pappalardo A.M., Federico C., Sabella G., Saccone S., Ferrito V. (2015). A COI Nonsynonymous Mutation as Diagnostic Tool for Intraspecific Discrimination in the European Anchovy *Engraulis encrasicolus* (Linnaeus). PLoS ONE.

[24] Ivanova N.V., Zemlak T.S., Hanner R.H., Hebert P.D.N. (2007). Universal primer cocktails for fish DNA barcoding. Mol. Ecol. Resour.

[25] Messing J. (1983). New M13 vectors for cloning. Meth. Enzymol..

[26] Schmidt T.R., Gold J.R. (1993). Complete Sequence of the Mitochondrial Cytochrome b Gene in the Cherryfin Shiner, *Lythrurus roseipinnis* (Teleostei: Cyprinidae). ASIH.

[27] Katoh K., Rozewicki J., Yamada K.D. (2019). MAFFT Online Service: Multiple Sequence Alignment, Interactive Sequence Choice and Visualization. Brief. Bioinform..

[28] Rozas J., Ferrer-Mata A., Sanchez-DelBarrio J.C., Guirao-Rico S., Librado P., Ramos-Onsins S.E., Sanchez-Gracia A. (2017). DnaSP 6: DNA Sequence Polymorphism Analysis of Large Data Sets. Mol. Biol. Evol..

[29] Kumar S., Stecher G., Li M., Knyaz C., Tamura K. (2018). MEGA X: Molecular Evolutionary Genetics Analysis across Computing Platforms. Mol. Biol. Evol..

[30] Kosakovsky Pond S.L., Frost S.D.W. (2005). Datamonkey: Rapid Detection of Selective Pressure on Individual Sites of Codon Alignments. Bioinformatics.

[31] Murrell B., Moola S., Mabona A., Weighill T., Sheward D., Kosakovsky Pond S.L., Scheffler K. (2013). FUBAR: A Fast, Unconstrained Bayesian AppRoximation for Inferring Selection. Mol. Biol. Evol..

[32] Murrell B., Wertheim J.O., Moola S., Weighill T., Scheffler K., Kosakovsky Pond S.L. (2012). Detecting Individual Sites Subject to Episodic Diversifying Selection. PLoS Genet..

[33] Silva G., Lima F.P., Martel P., Castilho R. (2014). Thermal Adaptation and Clinal Mitochondrial DNA Variation of European Anchovy. Proc. R. Soc. B.

[34] Waterhouse A., Bertoni M., Bienert S., Studer G., Tauriello G., Gumienny R., Heer F.T., De Beer T.A.P., Rempfer C., Bordoli L. (2018). SWISS-MODEL: Homology Modelling of Protein Structures and Complexes. Nucleic Acids Res..

[35] Buchan D.W.A., Jones D.T. (2019). The PSIPRED Protein Analysis Workbench: 20 Years On. Nucleic Acids Res..

[36] Ferrito V., Maltagliati F., Mauceri A., Adorno A., Tigano C. (2003). Morphological and Genetic Variation in Four Italian Populations of *Lebias fasciata* (Teleostei, Cyprinodontidae). Ital. J. Zool..

[37] Triantafyllidis A., Leonardos I., Bista I., Kyriazis I.D., Stoumboudi M.T., Kappas I., Amat F., Abatzopoulos T.J. (2007). Phylogeography and Genetic Structure of the Mediterranean Killifish *Aphanius fasciatus* (Cyprinodontidae). Mar. Biol..

[38] Pappalardo A.M., Ferrito V., Messina A., Guarino F., Patarnello T., De Pinto V., Tigano C. (2008). Genetic structure of the killifish *Aphanius fasciatus* Nardo 1827 (Teleostei, Cyprinodontidae), results of mitochondrial DNA analysis. J. Fish Biol..

[39] Ferrito V., Pappalardo A.M., Canapa A., Barucca M., Doadrio I., Olmo E., Tigano C. (2013). Mitochondrial Phylogeography of the Killifish *Aphanius fasciatus* (Teleostei, Cyprinodontidae) Reveals Highly Divergent Mediterranean Populations. Mar. Biol..

[40] Buj I., Miočić-Stošić J., Marčić Z., Mustafić P., Zanella D., Mrakovčić M., Mihinjač T., Ćaleta M. (2015). Population Genetic Structure and Demographic History of *Aphanius fasciatus* (Cyprinodontidae: Cyprinodontiformes) from Hypersaline Habitats in the Eastern Adriatic. Sci. Mar..

[41] Cavraro F., Malavasi S., Torricelli P., Gkenas C., Liousia V., Leonardos I., Kappas I., Abatzopoulos T.J., Triantafyllidis A. (2017). Genetic Structure of the South European Toothcarp *Aphanius fasciatus* (Actinopterygii: Cyprinodontidae) Populations in the Mediterranean Basin with a Focus on the Venice Lagoon. Eur. Zool. J..

[42] Langeneck J., Englezou C., Di Maggio M., Castelli A., Maltagliati F. (2021). Phylogeography of *Aphanius fasciatus* (Osteichthyes: Aphaniidae) in the Mediterranean Sea, with a Focus on Its Conservation in Cyprus. Hydrobiologia.

[43] Betts M.J., Russell R.B., Barnes M.R., Gray I.C. (2003). Amino Acid Properties and Consequences of Substitutions. Bioinformatics for Geneticists.

[44] Ward R.D., Holmes B.H. (2007). An analysis of nucleotide and amino acid variability in the barcode region of cytochrome c oxidase I (*cox1*) in fishes. Mol. Ecol. Notes.

[45] Degli Esposti M., De Vries S., Criml M., Ghelh A., Patarnello T., Meyer A. (1993). Mitochondrial Cytochrome b: Evolution and Structure of the Protein. Biochim. Biophys. Acta (BBA)-Bioenerg..

[46] McClellan D.A., Palfreyman E.J., Smith M.J., Moss J.L., Christensen R.G., Sailsbery J.K. (2004). Physicochemical Evolution and Molecular Adaptation of the Cetacean and Artiodactyl Cytochrome b Proteins. Mol. Biol. Evol..

[47] Beckstead W.A., Ebbert M.T.W., Rowe M.J., McClellan D.A. (2009). Evolutionary Pressure on Mitochondrial Cytochrome b Is Consistent with a Role of CytbI7T Affecting Longevity during Caloric Restriction. PLoS ONE.

[48] Dalziel A.C., Moyes C.D., Fredriksson E., Lougheed S.C. (2006). Molecular Evolution of Cytochrome c Oxidase in High-Performance Fish (Teleostei: Scombroidei). J. Mol. Evol..

[49] Baltazar-Soares M., de Araújo Lima A.R., Silva G. (2021). Targeted Sequencing of Mitochondrial Genes Reveals Signatures of Molecular Adaptation in a Nearly Panmictic Small Pelagic Fish Species. Genes.

[50] Chung D.J., Bryant H.J., Schulte P.M. (2017). Thermal acclimation and subspecies-specific effects on heart and brain mitochondrial performance in a eurythermal teleost (*Fundulus heteroclitus*). J. Exp. Biol..

