# Supplementary figures and images for: Evidence for Selection on Mitochondrial OXPHOS Genes in the Mediterranean Killifish Aphanius fasciatus Valenciennes, 1821
Source: Biology (Basel). 2024 Mar 25;13(4):212. doi: 10.3390/biology13040212 (PMC11048645; doi:10.3390/biology13040212)

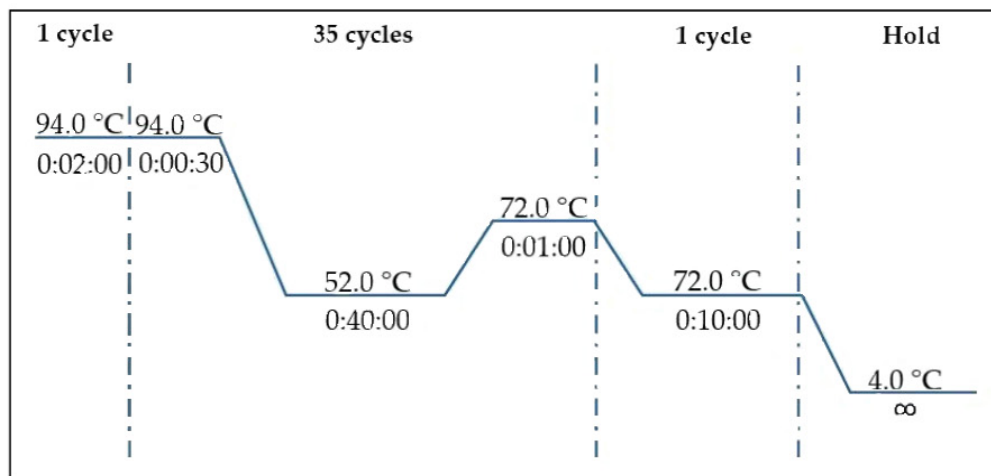

*COI*

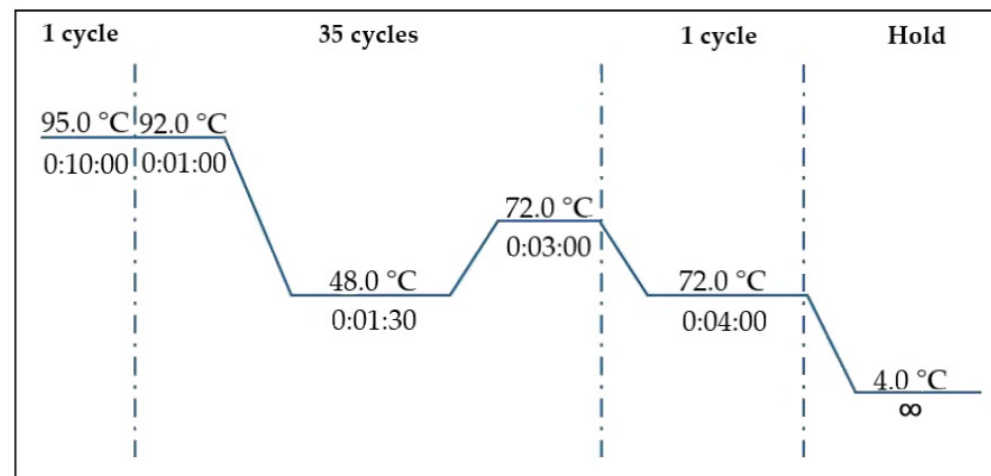

*Cytb*

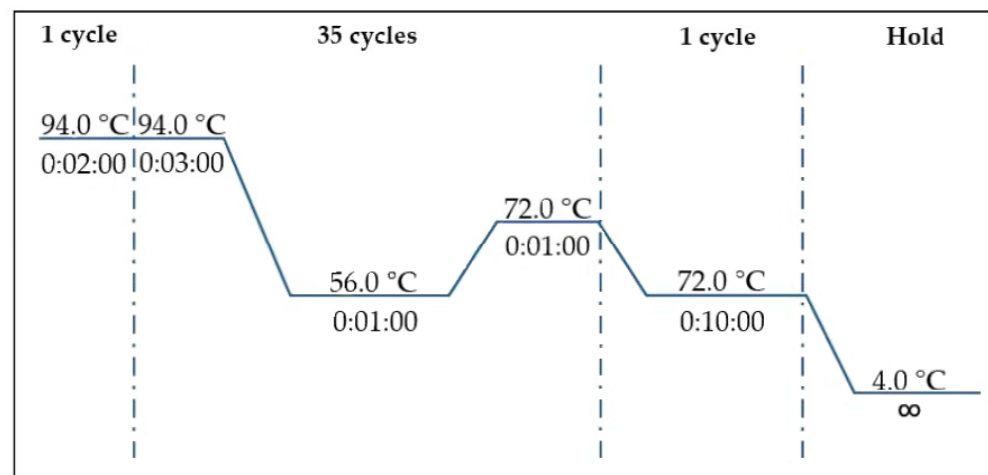

*ND1*

Supplement: Supplementary file 1 [file biology-13-00212-s001.zip › Supplementary Material/Supplementary Material Figure S1.pdf]
